# When Should ‘Clever’ Cheetah Breed? Seasonal Variability in Prey Availability and Its Effect on Cheetah Reproductive Patterns

**DOI:** 10.1002/ece3.71655

**Published:** 2025-06-24

**Authors:** Eleesha Annear, Liaan Minnie, Vincent van der Merwe, Graham I. H. Kerley

**Affiliations:** ^1^ Department of Zoology Centre for African Conservation Ecology, Nelson Mandela University Gqeberha South Africa; ^2^ School of Biology and Environmental Sciences University of Mpumalanga Mbombela South Africa; ^3^ Endangered Wildlife Trust Gauteng South Africa

**Keywords:** breeding season, cheetah, demographic‐specific predation, reproductive phenology, reproductive strategy

## Abstract

Breeding is energetically demanding for female mammals, with maternal and cub nutrition playing a major role in reproductive phases like conception, gestation, lactation and weaning. To meet these demands, adaptations to seasonal shifts in food availability are expected. Some predators may shift prey selection seasonally, optimizing foraging during energetically costly periods. Cheetah, *Acinonyx jubatus,* prefer adult prey in the dry season when younger prey are scarce but switch to neonate and juvenile prey during the wet season, presumably to optimize foraging during gestation and lactation. Given the wide distribution of cheetah across seasonal (i.e., distinct wet and dry seasons) and aseasonal environments (rainfall throughout the year) and the associated shifts in availability of prey demographic classes, we hypothesized that seasonal prey availability in seasonal systems, but not aseasonal systems, influences the timing of cheetah reproductive phases. Based on the birth dates of cheetahs in seasonal (*n* = 142) and aseasonal (*n* = 106) rainfall areas, 58.5% of litters were conceived during the wet season, with 60.6% born in the dry season. In contrast, aseasonal areas showed no seasonality in birth dates. Cheetah reproduction in seasonal environments is driven by the availability of neonate and juvenile prey, with conception and cub independence aligning with peaks in easy‐to‐catch neonates, while lactation coincides with the availability of larger juveniles. Although cheetahs are often viewed as specialized predators with limited ability to adapt to local environmental conditions, our findings suggest they can adjust reproductive patterns in response to prey availability. This adaptability is important as it will allow cheetahs to successfully raise cubs in the face of changing prey reproductive patterns in response to climate change.

## Graphical abstract

We develop and test the hypothesis that the seasonal availability of preferred prey demographic classes, like neonates and juveniles, drives cheetah reproductive patterns in seasonal systems. Cheetah reproductive cycles are synchronized with prey reproductive cycles in seasonal systems, allowing for key cheetah reproductive phases with high energy requirements to coincide with high availability of preferred easy‐to‐catch prey. Highlighting that cheetahs are able to adapt to local environmental conditions, and adjust their reproductive patterns to exploit that of their prey species.

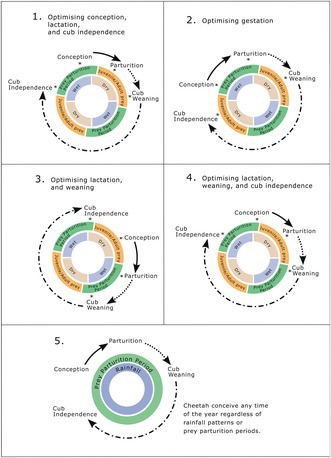

## 1 Introduction

Pre‐ and postpartum reproductive phases are energetically taxing for female mammals (Sadleir 1969; Ginther et al. 2024), with maternal nutrition playing a major role in conception (Robinson 1990; Simard et al. 2014), gestation (Matthiesen et al. 2010), parturition and lactation (Laurenson 1995; Kumon et al. 2010). Maternal nutrition is influenced by food availability and hunting success (East et al. 2015). This can reflect cycles of productive (high food availability in the wet seasons) and less productive (low food availability in the dry seasons) periods, which serve as strong ultimate selective pressures (sensu Davies et al. 2012) shaping the annual timing of female reproduction events. Thus, if the availability of preferred prey is constant across the year (e.g., reflecting aseasonal rainfall) predators are not expected to have a pronounced breeding season, as they are able to access optimal prey throughout the year (Anderson and Lovallo 2003; Pierce and Bleich 2003). However, if the availability of a predator's preferred prey fluctuates seasonally—e.g., fluctuating prey species abundance (Kjellander and Nordström 2003; Sundell et al. 2003; McKinnon et al. 2014; Spencer et al. 2017; Moorhouse‐Gann et al. 2020; Vettorazzi et al. 2022) or fluctuating prey demographic class abundance (Molinari‐Jobin et al. 2004; Owen‐Smith 2008; Makin and Kerley 2016; Annear et al. 2023)—the ‘clever’ predator (i.e., an optimally foraging predator; Owen‐Smith and Novellie 1982; Maré et al. 2021) should adjust its reproductive cycle so that energetically taxing reproductive phases coincide with periods of peak optimal prey availability (MacArthur and Pianka 1966; Charnov 1976).

Seasonal rainfall is associated with increased forage availability during the wet season (Giridhar and Samireddypalle 2015; Staver et al. 2021; Nketsang et al. 2025). This abundance in food availability has been shown to drive the timing of parturition in ungulates living in an environment with seasonal rainfall, with young being born during the wet season (Ogutu et al. 2014, 2015), typically between November and February in the southern hemisphere. As cheetah have been shown to select for neonate prey (Annear et al. 2023), this period of peak optimal prey availability would thus occur during the wet season, when their preferred prey are being born.

Here, we use the cheetah ( *Acinonyx jubatus* ) as a model to test if reproductive timing in predators corresponds to seasonal fluctuation in the availability of preferred prey demographic classes. Cheetahs represent the ideal model species, as access to prey is limited by their smaller body size (Clements et al. 2014). In addition, cheetahs display clear seasonal shifts in demographic‐specific prey preferences, corresponding to the seasonality in prey reproductive phases (Annear et al. 2023). Specifically, cheetahs prefer adults (27.8%) and subadults/juveniles (72.2%) and no neonates in the dry season, switching to preferring neonates (36.2%), fewer adults (12.8%), and subadults/juveniles (51.1%) in the wet season (Annear et al. 2023). Accordingly, we hypothesize that the timing of cheetah reproductive phases will be aligned with this seasonal pulse of optimal prey demographic classes, and hence also contribute to understanding the evolutionary ecology and adaptability of cheetahs to varying resource availability.

## 2 Cheetah Reproduction

Successful reproduction comprises a series of sequential and linked processes—i.e., conception, gestation, lactation and weaning—that vary depending on the focal predator and site‐specific variation in seasonal prey availability. Adaptive pressures apply to all stages, with adaptive shifts in the timing of one reproductive phase (i.e., synchronization with resource pulses) leading to shifts in the timing of the following phases. Given our focus on cheetahs, we present a brief overview of the reproductive cycle and highlight reproductive timing predictions related to seasonal variation in the availability of different prey demographic classes.

Cheetah females are polyestrous, with an oestrous cycle that is 13.6 ± 1.2 days long (Brown et al. 1996). They are induced ovulators that are able to conceive throughout the year, with a gestation period of approximately 3 months (Brown 2011; Kelly et al. 1998; Crosier et al. 2022). Cheetah cubs start consuming meat provided by their mother at 32 days old (Langer 2008) but will nurse until they are weaned at 4 months of age (Laurenson 1993). Mothers introduce live prey to cubs at 2.5–3.5 months of age, but cubs only start suffocating practice prey at 4.5–6.5 months old (Caro and Hauser 1992). Cubs typically become independent at 18 months old (Caro 1994). Therefore, this reproductive cycle is approximately 21 months long.

Lactation is considered the most energetically taxing reproductive phase in mammals (Sadleir 1969; Oftedal and Gittleman 1989; East et al. 2015). However, recently Ginther et al. (2024) have shown that the indirect energetic costs (i.e., conception and gestation) of reproduction are far higher than the direct energetic costs (i.e., lactation). Thus, cheetahs may be more likely to optimize the conception and gestation phases.

## 3 Predictions

Due to the sequential nature of the reproductive phases in mammals, the onset of conception (and the following reproductive phases) may be driven by optimizing prey selection during a specific reproductive phase, leading to an adjustment in the onset of the other reproductive phases. As such, we develop several predictions that may not be mutually exclusive but highlight the reproductive phase where cheetahs optimize prey selection, and thus drive the entire reproductive cycle.

**FIGURE 1:**
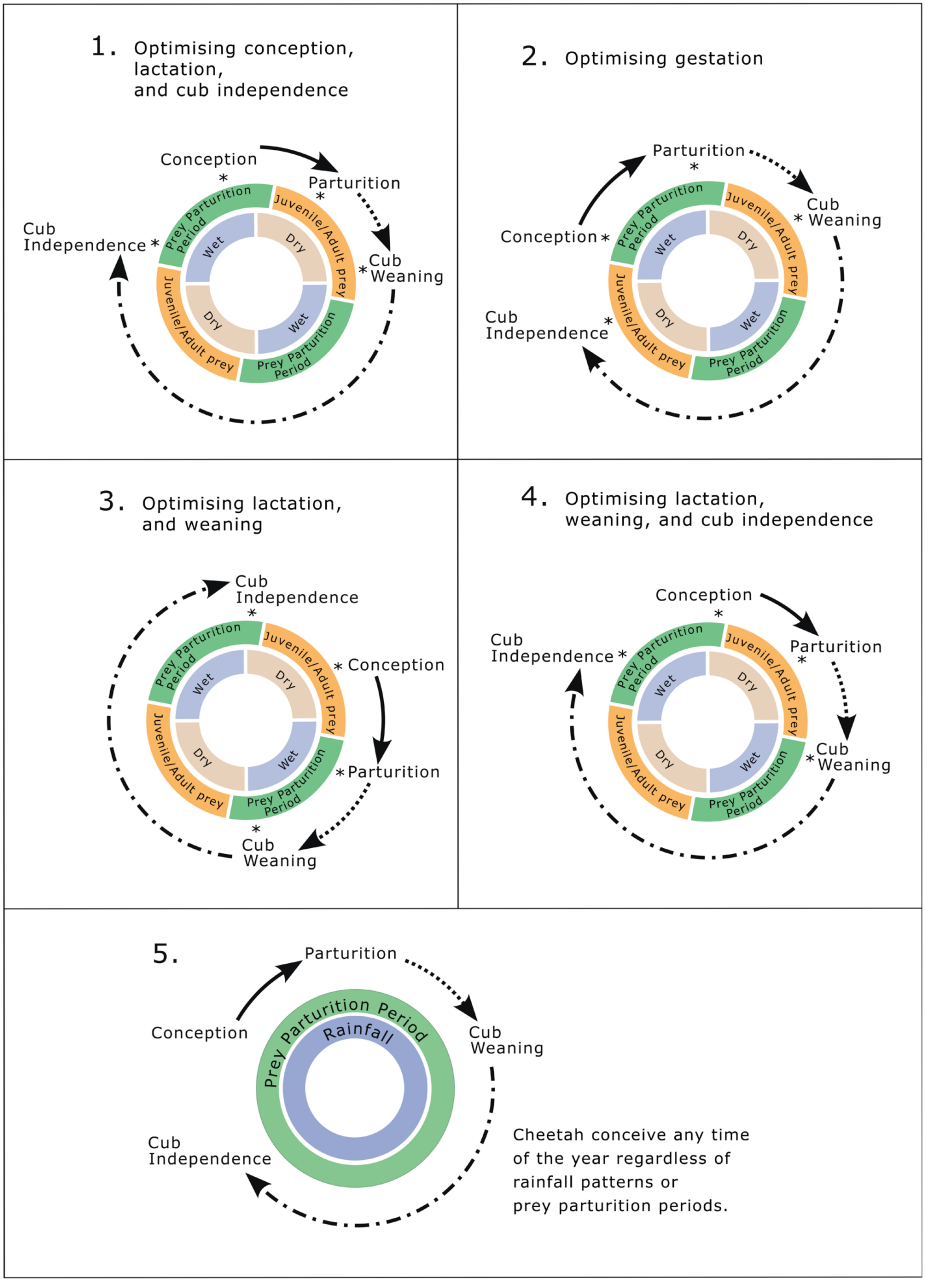
Predicted pattern (see text for detail) of cheetah reproduction in relation to preferred prey availability and rainfall over a 2‐year period (reflecting the conception to independence timing for cheetahs). Predictions 1–4 illustrate patterns of cheetah reproduction in a seasonal system (with a distinct wet and dry season, and therefore a distinct prey breeding season) where they optimize different reproductive phases. The point of the reproductive phase is indicated by a star (*). The solid line indicates the gestation period, and the dotted line the lactation period. Prediction 5 represents the alternative hypothesis where cheetah, in aseasonal environments, will not optimize any specific reproductive phase and consistently breed throughout the year irrespective of rainfall seasonality or prey breeding seasons. Note that Predictions 1 and 4 are nearly coincidental. Neonate prey (< 3 months old) are available to hunt during the prey parturition period (green). In seasonal environments, most prey species give birth to young during the wet season (November–February in the southern hemisphere). As the prey breeding season progresses, juvenile prey (3–12 months old) abundance increases, as neonates mature into juveniles. Thus, during the dry season, juvenile or adult (> 12 months) prey are available to hunt.

1. Cheetah conception will occur towards the end of the prey parturition period when cheetah body condition is elevated (Figure 1), owing to the availability of easy‐to‐catch juvenile prey. Cheetah parturition and lactation will accordingly occur at the start of the dry season when only juvenile and adult prey are available (Figure 1). Therefore, cheetah may hunt larger juveniles and adults during lactation when females need to increase their food intake until their cubs are weaned at the end of the dry season. Cub weaning will occur at the end of the dry season, with cub independence coinciding with the start of the following prey parturition period (Figure 1). This timing optimizes prey availability for conception, lactation and cub independence.
2. Cheetahs will conceive at the start of the prey parturition period when the abundance of easy‐to‐catch prey demographic classes is increasing, and therefore gestation occurs when neonate prey are at peak abundance (Figure 1). Cheetah parturition will occur towards the end of the prey parturition period, with lactation occurring as juvenile prey are increasing in abundance (Figure 1). Weaning and the subsequent independence of cubs will occur during the dry season. This timing optimizes prey availability for gestation.
3. Cheetahs will conceive during the dry season, which will lead to late gestation and parturition coinciding with the start of the prey parturition period (Figure 1). Therefore, lactation and weaning will occur during the peak of the prey parturition period, and cubs will become independent at the start of the dry season (Figure 1). This timing optimizes prey availability for lactation and weaning.
4. Conception will occur at the end of the prey breeding season, with cheetahs giving birth during the dry season. As such, lactation will occur when juvenile prey are older than 7 months. Cub weaning and female provisioning of practice prey will coincide with the start of the prey breeding season and cub independence will occur at the start of the following prey breeding season (Figure 1). This timing optimizes prey availability for lactation, weaning, and independence.
5. Alternatively, cheetahs will show no seasonality in their reproduction and reproduce at any time throughout the year in areas with seasonal rainfall. In seasonal systems, this indicates a lack of adaptive response to prey availability. This pattern should mimic reproductive timing in areas with aseasonal rainfall, where neonate and juvenile prey are potentially available throughout the year (Figure 1).

## 4 Methods

### 4.1 Study Sites

**FIGURE 2:**
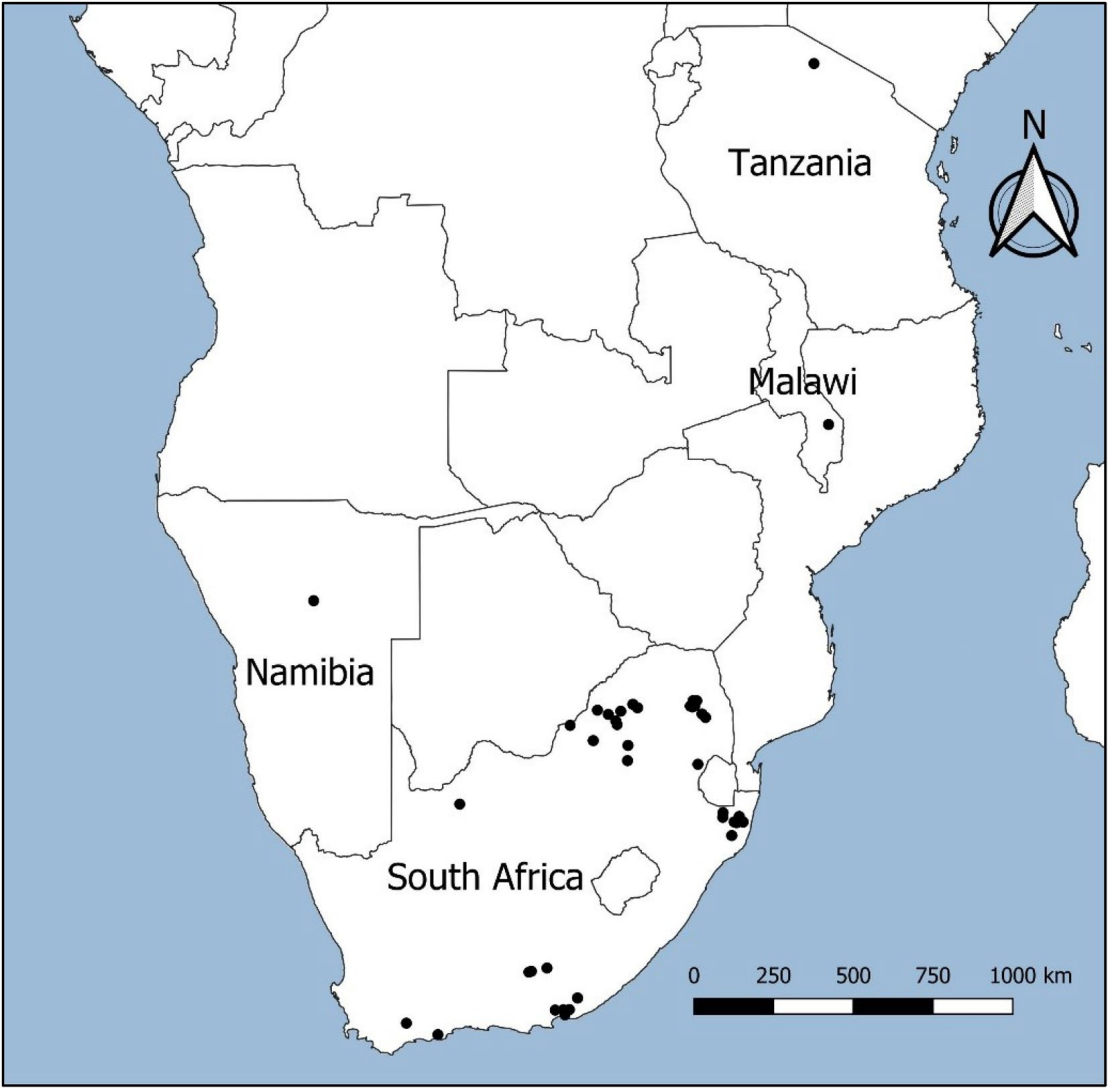
Sites with cheetahs (solid circles) where data on reproduction was obtained.

Cheetah breeding records were acquired from the Endangered Wildlife Trust's (EWT) cheetah metapopulation project (V. van der Merwe, Unpubl. data) and the literature (Laurenson et al. 1992; Marker et al. 2003; Bissett and Bernard 2011). These data include sites in fenced reserves across South Africa, unfenced reserves in Malawi and Tanzania, and farmland in Namibia (Figure 2). These sites occur in four different biomes, including the Savanna (*n* = 30), Grassland (*n* = 2), Albany thicket (*n* = 8), and Fynbos (*n* = 2; see Table S1 for detailed site information).

Rainfall has a significant influence on the breeding season of prey species (Ogutu et al. 2014; Morin et al. 2021). Therefore, using rainfall data obtained from WorldClim (Fick and Hijmans 2017), sites were categorized as seasonal or aseasonal. Seasonal sites display distinct peaks and valleys in the average monthly rainfall received over a year. In contrast, areas with aseasonal rainfall experience rainfall throughout the year and thus lack the distinct peaks and valleys of rainfall in seasonal areas. We used a two‐way ANOVA to test if there was a significant difference in the mean monthly rainfall received in seasonal and aseasonal sites. The aseasonal sites received significantly lower rainfall than the seasonal sites from November to March but received higher rainfall from April to October (ANOVA: *F*_11(456)_ = 15.250, *p* < 0.001). Prey breeding seasons, associated with prevailing rainfall patterns, were determined for each site using the available literature (Fairall 1968; Skinner and Chimimba 2005; Kingdon et al. 2013; Ogutu et al. 2014), with most prey species giving birth to young between November–February in the southern hemisphere.

### 4.2 Cheetah Reproductive Phases

We estimated the number of observed litters and litter size for the major reproductive phases including conception, parturition, weaning, and cub independence as follows. For each observed litter at each site in the dataset, we extracted the month of cub emergence from the den, as well as litter size. However, as the dataset is based on observations of litters, we applied a correction factor of 3 weeks to the first recorded sighting of a litter to estimate parturition month. This is because cheetah cubs are hidden in the birth den site for approximately 10–14 days (Laurenson 1993), but become more conspicuous after 3 weeks as the female moves her cubs more frequently (Lindeque and Skinner 1982; Kingdon and Hoffman 2013). The month of conception was estimated by subtracting the cheetah gestation period of 3 months (Laurenson et al. 1992) from the parturition month. The month of weaning and independence of each litter was estimated by adding the age of weaning (4 months) and independence (18 months) to the parturition month. Weaning and independence data were not available for the populations in Tanzania and Namibia (Laurenson et al. 1992; Laurenson 1993; Marker et al. 2003).

### 4.3 Statistical Analyses

Due to the cyclical nature of reproduction, we used a Rayleigh test to determine if the reproductive phase data (i.e., number of litters conceived, born, weaned, or reaching independence) were non‐uniformly distributed across a 1‐year period for seasonal, aseasonal, and pooled sites. This allowed us to test the predictions that reproductive phase data will cluster around a mean direction coinciding with wet and dry periods, as well as the associated availability of prey demographic classes (Figure 1).

Generalized linear models (GLM) with a Poisson distribution and logit link function (Quinn and Keough 2002) were used to determine the influence of rainfall and prey breeding season on the number of litters in each of the cheetah's reproduction phases (i.e., conception, parturition, weaning, and cub independence). Predictor variables were tested for collinearity by calculating variance inflation factors (VIFs). A VIF value exceeding 2.5 was considered indicative of collinearity (Midi et al. 2010; Senaviratna and Cooray 2019). The predictor variables had a VIF value below the cut‐off point of 2.5, indicating that they are not collinear.

The homogeneity of variances was tested using Levene's test for categorical variables and Fligner–Killeen's test for continuous variables. The predictor variables had equal variances for the cheetah conception, parturition, weaning and independence models. The most parsimonious model was selected using AIC (Akaike information criterion) before ΔAICc for small sample sizes (ΔAICc; Burnham and Anderson 2002) to select the top models (≤ 2 ΔAICc). Model coefficients were estimated using model averaging (Guthery et al. 2003; Symonds and Moussalli 2011) using the package ‘MuMIn’ in R (Barton and Anderson 2002). All statistical analyses were conducted in the programme R (R Core Team 2021).

## 5 Results

### 5.1 The Influence of Rainfall Seasonality on Cheetah Reproduction

Cheetahs could conceive and produce litters throughout the year, irrespective of rainfall seasonality (*n* = 246 litters). There was no significant difference in the number of litters conceived (*F*_1(130)_ = 2.124, *p* = 0.147) and born (*F*_1(130)_ = 2.124, *p* = 0.147) between sites with seasonal and aseasonal rainfall. In contrast, the number of litters weaned (*F*_1(106)_ = 11.56, *p* < 0.001) and that were able to reach independence (*F*_1(106)_ = 8.364, *p* = 0.004) was significantly higher in sites with aseasonal rainfall.

**FIGURE 3:**
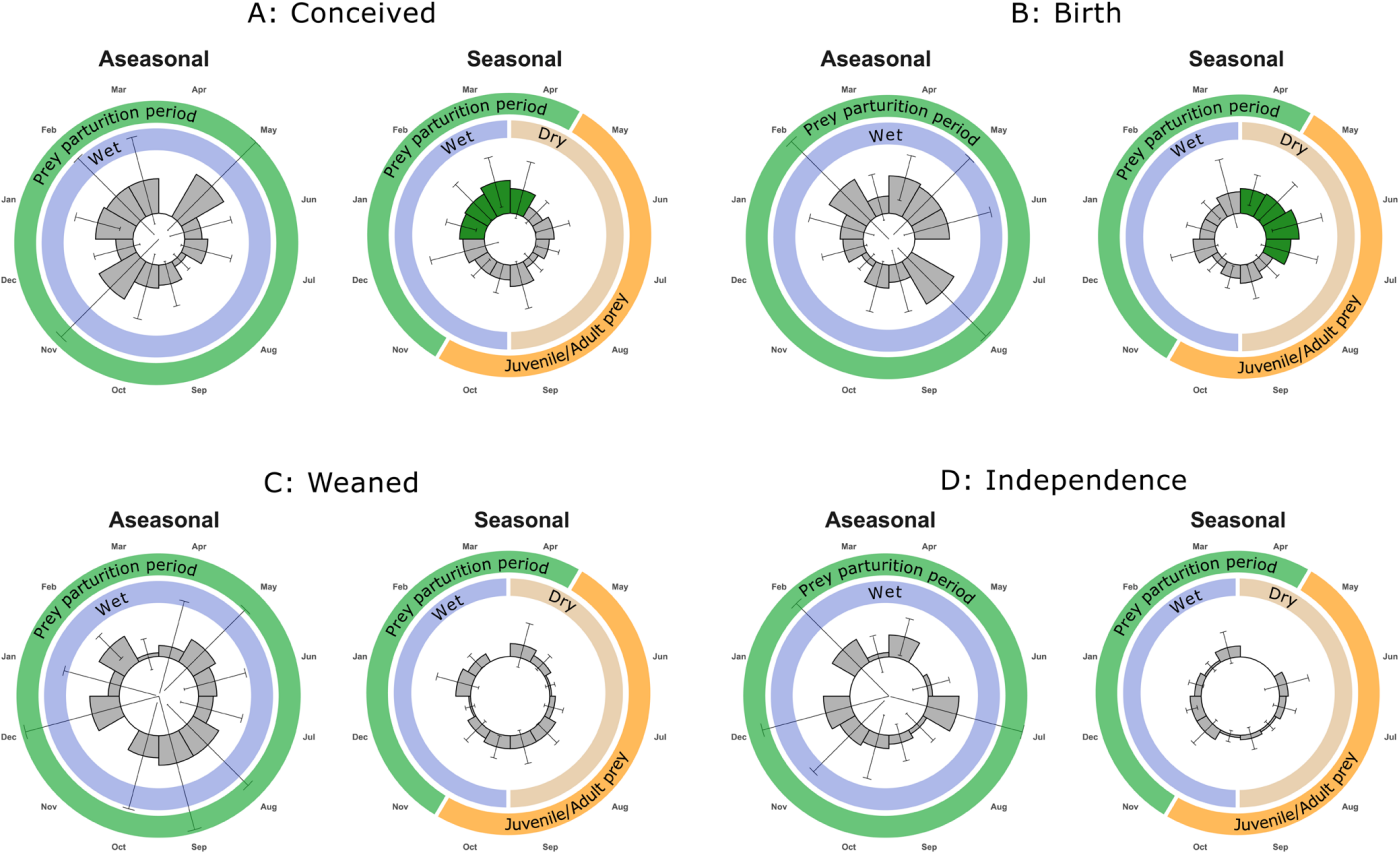
Radar plots show the mean number of cheetah litters (±95% CI) conceived (A), born (B), weaned (C), and becoming independent (D) in sites with seasonal and aseasonal rainfall during a 1‐year period. The significant peaks are indicated by dark green bars. The outer circle indicates when neonate prey are available during the prey parturition period (green) and when juvenile and adult prey are available (orange). The inner circle indicates the rainfall season (wet: Blue; dry: Light orange).

In populations with seasonal rainfall (*n* = 142 litters), conceptions peaked between January and April (Rayleigh statistic = 0.201, *p* = 0.004, 46.5% of conceptions; Figure 3), with the corresponding peak in the number of litters born between April–July (Rayleigh statistic = 0.201, *p* = 0.004, *n* = 142, Figure 3). These results support predictions one and four (Figure 1). In populations with seasonal rainfall, 58.5% of the conceptions occurred during the wet season (Figure 3), with the majority of births occurring during the dry season (60.6%; Figure 3). Cheetahs weaned (Rayleigh statistic = 0.144, *p* = 0.319, *n* = 55 litters) their litters, and the litters became independent (Rayleigh statistic = 0.195, *p* = 0.323, *n* = 30 litters) throughout the year in areas with seasonal rainfall. During the wet season, 66.6% of litters reached independence.

Cheetah conception (Rayleigh statistic = 0.146, *p* = 0.105, *n* = 106 litters), parturition (Rayleigh statistic = 0.146, *p* = 0.105, *n* = 106), weaning (Rayleigh statistic = 0.115, *p* = 0.445, *n* = 61), and independence (Rayleigh statistic = 0.109, *p* = 0.655, *n* = 36) occurred throughout the year in sites with aseasonal rainfall (Figure 3). This suggests that cheetahs occurring in areas with aseasonal rainfall do not show any seasonality in their reproduction (Prediction 5; Figure 1). Furthermore, the relatively wider confidence intervals at aseasonal sites highlight a greater variability in the timing of weaning (*F*_1(22)_ = 10.89, *p* = 0.003) and independence (*F*_1(22)_ = 9.65, *p* = 0.005) of cheetah cubs in aseasonal rainfall areas compared to seasonal rainfall areas.

### 5.2 The Influence of Rainfall and Prey Breeding Season on Cheetah Reproduction

**FIGURE 4:**
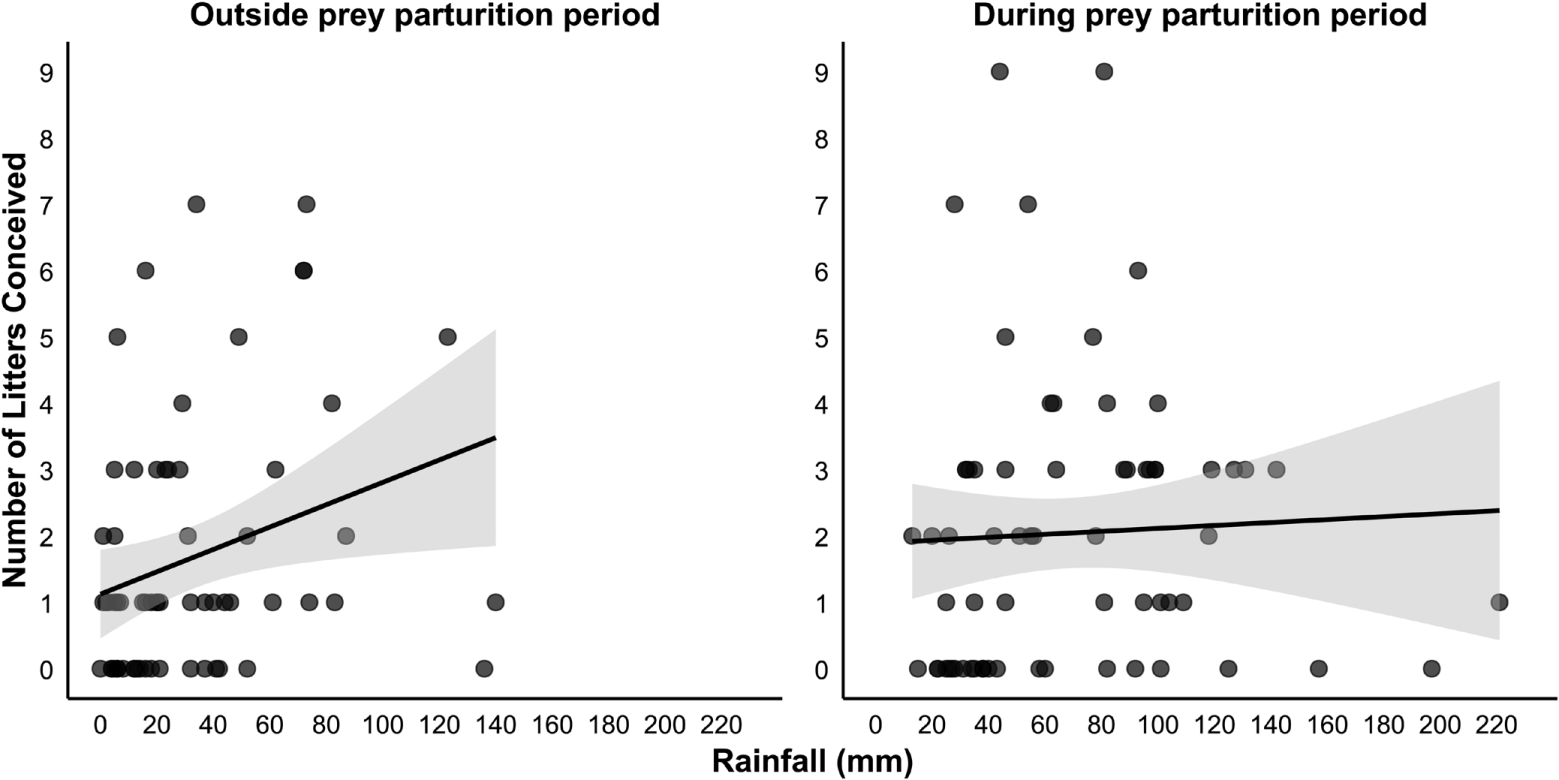
The relationship between prey parturition period and rainfall and its influence on cheetah conception.

The top model for cheetah conception (weight of evidence (*w*) = 63%) included rainfall and prey breeding season with an interaction between rainfall and prey breeding season (Conception model 1, Table 1). Additionally, Conception model 2 (ΔAICc = 1.09, *w* = 37%; Table 1) was also supported. The averaged model indicated that cheetahs conceived significantly more litters with an increase in rainfall (model‐averaged coefficient = 0.006 ± 0.003, *z* = 2.19, *p* = 0.02) and during the prey breeding season (model‐averaged coefficient = 0.434 ± 0.215, *z* = 1.98, *p* = 0.04), as per predictions one and four (Figure 1). Further, the interaction between rainfall and prey breeding season was significant (model‐averaged coefficient = −0.007 ± 0.003, *z* = 2.26, *p* = 0.02), with cheetah conception increasing significantly (slope = 0.017) as rainfall increased outside prey parturition periods (Figure 4), which indicates that increased rainfall may act as a cue for cheetah to start conceiving. In contrast, within prey parturition periods, cheetah conception was not influenced by rainfall (slope = 0.002; Figure 4).

**TABLE 1:** The top Generalized Linear Models explaining the variation in cheetah reproductive phases (conception [C], parturition [P], weaning [W], independence [I]) based on model selection using Akaike Information Criteria for small sample sizes (ΔAICc; Burnham and Anderson 2002). Results include model degrees of freedom (df), ΔAICc representing the difference in relation to the top model, and the model weight (*w*). The variables included in the models are rainfall (R), and prey breeding season (PBS), with: Representing an interaction between the two.

Model	Description	df	AICc	ΔAICc	Loglikelihood	*w*
*Conception*						
1	C ~ *R* + PBS + *R*: PBS	4	541.30	0.00	−266.50	0.63
2	C ~ *R*	2	542.40	1.09	−269.15	0.37
*Parturition*						
1	*P* ~ PBS	2	544.50	0.00	−270.24	0.72
2	*P* ~ PBS + *R*	3	546.40	1.88	−270.13	0.28
*Weaning*						
1	W ~ PBS	2	330.45	0.00	−163.17	0.69
2	W ~ PBS + *R*	3	332.04	1.60	−162.91	0.31
*Independence*						
1	I ~ PBS	2	238.90	0.00	−117.43	0.72
2	I ~ PBS + *R*	3	240.80	1.89	−117.31	0.28

The top models (Table 1) for parturition, cub weaning, and cub independence had the same structure, with prey breeding season being the only predictor variable included in the best models (*w* range across models: 0.69–0.72). However, across all response variables, both prey breeding season and rainfall were included in the second‐best models (ΔAICc range across models: 1.6–1.89; *w* range: 0.28–0.31; Table 1). The model‐averaged coefficients indicated that (1) cheetah parturitions were significantly lower during the prey breeding season (model‐averaged coefficient = −0.297 ± 0.133, *z* = 2.21, *p* = 0.03), (2) weaning occurred during the prey breeding season (model‐averaged coefficient = 0.595 ± 0.208, *z* = 2.818, *p* = 0.004), and (3) most cheetah cubs became independent during the following prey breeding season (model‐averaged coefficient = 0.901 ± 0.245, *z* = 3.02, *p* = 0.002).

## 6 Discussion

In this study, we show that cheetah optimize their reproductive pattern to align with pulses of prey availability, as hypothesized. In seasonal systems, the multi‐site analyses show that cheetah conception typically occurs towards or at the end of the prey parturition period, with cheetahs giving birth during the dry season. Litters are therefore typically weaned during the prey parturition period and litter independence coincides with the following prey parturition period when the abundance of easy‐to‐catch neonate prey is high. This reproductive timing in cheetah reproductive phases relative to prey reproductive phases supports prediction four (Figure 1). Importantly, cheetah reproductive timing appears to be driven predominantly by weaning litters within the prey parturition period, given that this produced the most significant response across all the models. These results are novel and highlight the importance of preferred prey (i.e., neonate prey; Annear et al. 2023) for the timing of cheetah reproduction, as well as for litters to reach g independence successfully.

Cheetah reproduction, from mating and conception to cub independence, has been described in detail in the literature (Laurenson et al. 1992; Caro 1994; Marker et al. 2003; Bissett and Bernard 2011; Mills and Mills 2017). However, the majority of studies were conducted at one site, with small sample sizes. Furthermore, these studies only described seasonality in cheetah conception and parturition without addressing the potential drivers of reproductive timing, including the seasonal availability of preferred prey demographic classes. Here, we have bridged that gap to include cub weaning and independence across multiple sites. While we have successfully linked adaptations of cheetah life history timing to that of their prey in seasonal systems, we have not explored the mechanisms behind these adaptive responses. Given the evidence provided here of the alignment of cub independence with neonate prey availability, we hypothesize that the proximate mechanism lies in the area of the development of hunting behaviour of cubs, rather than the physiological benefits of increased nutrition for the mother.

### 6.1 Seasonality and Conception

If a predator is resource‐limited, an increase in prey availability during the prey breeding season should lead to an increase in reproductive output (Giesel 1976). Furthermore, individuals with access to high‐quality food resources often attain better body condition, which increases reproduction (Parker et al. 2009). Prey availability influences oestrus in lions *Panthera leo* , with lions coming into oestrus more often when prey are abundant compared to when prey are scarce (Packer et al. 1990). Similarly, increased conception has been linked to increased prey availability in other predators like the Eurasian lynx *Lynx lynx* and the Canada lynx *Lynx canadensis* (Brand and Keith 1979; Pulliainen et al. 1995), coyote *Canis latrans* (Todd and Keith 1983), some species of boreal forest owls (Lehikoinen et al. 2011), and black‐backed jackals *Canis mesomelas* (Minnie et al. 2016).

Cheetahs' preference for and consumption of neonate and juvenile prey can shift seasonally, as the availability of neonate and juvenile prey fluctuates throughout the year (Annear et al. 2023). Here, the varying timing of cheetah conception in areas with differing rainfall seasonality has been linked to the fluctuating availability of preferred demographic classes, which was originally suggested by Laurenson et al. (1992) and then Caro (1994). Caro (1994) suggested that the evidence for an association between the timing of cheetah conception and neonate availability in the Serengeti was weak. However, this may have been due to the aseasonality in rainfall, with prey breeding cycles being less pronounced closer to the equator (Lindeque and Skinner 1982). At temperate latitudes, most species rely on photoperiod as a cue to time conception, with other cues like rainfall and food availability modulating the impact of photoperiod (Dardente et al. 2016). However, in this study, areas with aseasonal rainfall in temperate southern Africa showed similar cheetah conception patterns to areas close to the equator. This may be due to prey species breeding throughout the year in temperate southern African areas where there is forage available year‐round due to the aseasonal rainfall (Skinner et al. 2002; Ogutu et al. 2015), resulting in no definitive pulses in the availability of neonate and juvenile prey. In contrast, cheetah populations in seasonal environments in southern Africa synchronized conception to ensure that the birth of offspring coincided with the end of the parturition period of seasonally reproducing prey species. However, individual variation is apparent, which results in some individuals showing seasonal breeding and others breeding throughout the year (Bronson 2009). Thus, varying photo‐responsiveness (modulated by rainfall and food availability) may explain the varying cheetah conception between areas with seasonal and aseasonal rainfall. This was supported by the interactive relationship between rainfall and prey breeding season across our study populations (Figure 4). Cheetah conception increased with rainfall outside the prey parturition periods, with rainfall not impacting conception during the prey parturition period when the availability of easy‐to‐catch neonate prey was high. This indicates that increased rainfall may act as a cue for cheetahs to start conceiving as prey populations are likely to enter their parturition period (Ogutu et al. 2014), and preferred easy‐to‐catch neonate prey will increase in availability.

### 6.2 Seasonality and gestation, parturition, lactation, weaning, and cub independence

Lactation is one of the most energetically expensive activities during reproduction (Sadleir 1969). Prey availability can influence the growth rate of offspring (Hofer and East 1993), and carnivores have been shown to abandon litters when prey is scarce, and it is difficult to acquire food (Caro 1994). Female cheetahs must consume 1.5 kg of food per day to support themselves and their cubs while lactating (Laurenson 1995). It is therefore important for females to give birth during periods with high food availability. Ungulates have been shown to give birth during the wet season when there is a high abundance of forage (Ogutu et al. 2014) to support lactating females and their young. In seasonal systems, the majority of female cheetahs gave birth during the dry season after the prey breeding season. This is likely driven by the increase in easy‐to‐catch neonate prey during the gestational phase when female hunting is compromised owing to her increased body mass, in addition to the high energy cost of gestation (Ginther et al. 2024). Parturition and subsequent lactation typically occurred during the dry season when relatively larger juvenile prey are available. During this lactation phase, female hunting is no longer physically compromised, allowing females to hunt larger juvenile prey which offers a higher energetic reward (MacArthur and Pianka 1966; Charnov 1976) when energy is most needed (Sadleir 1969; Oftedal and Gittleman 1989; Laurenson 1995) and may have the greatest impact on the timing of cheetah reproduction. Prey may also be more reliably located close to water sources during the dry season, thus reducing the time spent by female cheetahs looking for prey. Female cheetahs, therefore, maximize their net energetic gain from the neonate and juvenile prey demographic classes during the gestation and lactation reproduction phases (MacArthur and Pianka 1966; Charnov 1976), enabling females to successfully wean their cubs. When cubs reach independence, they need to be able to hunt and feed themselves. Here it was shown that cub independence coincided with high neonate and juvenile availability during the prey breeding season. Although cheetah mothers are known to bring live practice prey to their cubs (Mills and Mills 2017), the hunting skills of cheetah cubs are weak up to and beyond independence (Caro 1994). Thus, access to easy‐to‐catch neonate prey may be vital to a newly independent cub's survival.

While our findings suggest a relationship between preferred prey availability (presence of neonates during the prey breeding season) and cheetah reproductive phases, we acknowledge that cheetah reproductive timing is likely influenced by a suite of interacting ecological factors. Photoperiod, rainfall, and other environmental variables can act as proximate cues that influence reproductive physiology and behaviour in felids, either independently or in combination with prey dynamics. In this study, we incorporated rainfall into our models to account for some of this complexity. Future research integrating long‐term hormonal monitoring, fine‐scale ecological data, and direct prey abundance metrics would help disentangle the relative contributions of these cues. Thus, rather than implying a singular cause‐effect mechanism, our findings support the idea that prey availability is one of multiple ecological drivers contributing to reproductive phenology in cheetahs.

Given that the data we used for estimating cheetah breeding patterns were derived from population data collected for other purposes, and that we had to estimate the timing of actual parturition, there is uncertainty as to specific breeding event dates. In addition, it is likely that birth events did occur in these populations, but the litters did not survive to be counted later (c.f., Mills and Mills 2017). If such failed litters were seasonally biased, then this would influence our data. Furthermore, the nature of the data means that our findings are correlative, however, they are clearly supportive of the hypothesis. These hypotheses and predictions may be further tested by evaluating expected shifts in the birth season of Northern hemisphere cheetah populations in Iran and India. Although these populations are both small and given the recent establishment of the Indian population from Southern hemisphere sources, it may take many generations for these breeding cycles to respond. However, this is the first study to relate seasonality in prey demographic class availability to timing of cheetah reproductive cycles and there is tremendous value in closely monitoring breeding events in species such as cheetah.

## 7 Conclusion: The ‘Clever’ Cheetah

Our results highlight two novel and important aspects of the ‘clever’ cheetah. Firstly, the availability of specific prey demographic classes (i.e., neonates and juveniles) drives cheetah reproduction patterns in seasonal systems. The phases of the cheetah reproduction cycle are synchronized with prey reproduction cycles such that: (1) Conception coincides with the peak availability of neonates to ensure that females have sufficient body condition to reproduce; (2) lactation coincides with the high availability of both neonates and juveniles to ensure that females obtain enough resources to sustain energetically costly lactation; and (3) Cub independence coincides with the peak availability of neonates to ensure that cubs separating from their mother have access to easy‐to‐catch neonate prey. Secondly, cheetahs are often perceived as highly specialized predators with a limited ability to adapt to local environmental conditions (Marker 2019). This limited ability to adapt apparently contributes, in part, to the small proportion of weaned litters and litters that reach independence (Laurenson et al. 1992) and thus exacerbates their threatened status (Durant et al. 2017). However, here we highlight that cheetahs are able to adjust their reproductive patterns to exploit that of their prey species. This adaptability is important as it will allow cheetahs to successfully raise cubs to independence in the face of changing prey reproductive patterns in response to climate change.

## Author Contributions

**Eleesha Annear:** conceptualization (supporting), data curation (lead), formal analysis (lead), funding acquisition (supporting), visualization (lead), writing – original draft (lead), writing – review and editing (equal). **Liaan Minnie:** conceptualization (equal), formal analysis (supporting), funding acquisition (equal), supervision (equal), writing – review and editing (equal). **Vincent van der Merwe:** data curation (supporting), writing – review and editing (equal). **Graham I. H. Kerley:** conceptualization (equal), funding acquisition (equal), supervision (equal), writing – review and editing (equal).

## Ethics Statement

The project was conducted with Nelson Mandela University animal ethics approval (A19‐SCI‐ZOO‐006).

## Conflicts of Interest

The authors declare no conflicts of interest.

## Supporting information


Appendix S1.

## Data Availability

All the required data are uploaded as Supporting Information.
