# Supplementary material for: When Should ‘Clever’ Cheetah Breed? Seasonal Variability in Prey Availability and Its Effect on Cheetah Reproductive Patterns
Source: Ecol Evol. 2025 Jun 24;15(6):e71655. doi: 10.1002/ece3.71655 (PMC12185933; doi:10.1002/ece3.71655)
Supplement: Supplementary file 1 — Appendix S1. [file ECE3-15-e71655-s001.docx]

Appendix A

Table A.1: The name and location of study sites where cheetah gave birth, including information on annual rainfall, rainfall seasonality (estimated using WorldClim rainfall data), and biome.

| **Study site** | **Co-ordinates** | **Country, Province** | **Average annual rainfall (mm)** | **Average rainfall October-March (mm)** | **Average rainfall April-September (mm)** | **Rainfall seasonality** | **Biome** |
| --- | --- | --- | --- | --- | --- | --- | --- |
| Entabeni Private Game Reserve | S24.2233, E28.6594 | South Africa, Limpopo | 686 | 103.12 | 12.41 | Seasonal | Savanna |
| Welgevonden Private Game Reserve | S24.3147, E28.0541 | South Africa, Limpopo | 650-900 | 80.54 | 8.91 | Seasonal | Savanna |
| Witwater Private Game Reserve | S24.0900, E28.4800 | South Africa, Limpopo | 588 | 92.85 | 10.63 | Seasonal | Savanna |
| Thaba Tholo Game Farm | S24.2811, E27.2319 | South Africa, Limpopo | 504 | 69.89 | 7.25 | Seasonal | Savanna |
| Mabula Private Game Reserve | S24.7333, E27.9269 | South Africa, Limpopo | 641 | 87.93 | 9.08 | Seasonal | Savanna |
| Marakele National Park & Pty | S24.3833, E27.6167 | South Africa, Limpopo | 556-630 | 95.21 | 9.18 | Seasonal | Savanna |
| Pilanesberg National Park | S25.2611, E27.1008 | South Africa, North West | 630 | 78.35 | 10.03 | Seasonal | Savanna |
| Dinokeng Game Reserve | S25.3800, E28.3805 | South Africa, Gauteng | 674 | 87.67 | 8.67 | Seasonal | Savanna |
| Rietvlei Nature Reserve | S25.8969, E28.2939 | South Africa, Gauteng | 724 | 97.13 | 13.73 | Seasonal | Grassland |
| Meletse Private Game Reserve | S24.6246, E27.8775 | South Africa, Limpopo | 574 | 92.73 | 10.07 | Seasonal | Savanna |
| Madikwe Conservancy | S24.7058, E26.4891 | South Africa, North West | 500 | 63.63 | 6.60 | Seasonal | Savanna |

Table A.1 Continued

| **Study site** | **Co-ordinates** | **Country, Province** | **Average annual rainfall (mm)** | **Average rainfall October-March (mm)** | **Average rainfall April-September (mm)** | **Rainfall seasonality** | **Biome** |
| --- | --- | --- | --- | --- | --- | --- | --- |
| Blue Canyon Conservancy | S24.5127, E31.0266 | South Africa, Limpopo | 721 | 98.85 | 18.20 | Seasonal | Savanna |
| Karongwe Private Game Reserve | S24.1766, E30.5516 | South Africa, Limpopo | 586 | 82.93 | 16.09 | Seasonal | Savanna |
| Makutsi Private Game Reserve | S24.1461, E30.5016 | South Africa, Limpopo | 633 | 84.59 | 17.49 | Seasonal | Savanna |
| Nkomazi Private Game Reserve | S26.0005, E30.7577 | South Africa, Mpumalanga | 905 | 115.74 | 17.38 | Seasonal | Savanna |
| Pidwa Wilderness and Makali Private Game Reserve | S24.1688, E30.6583 | South Africa, Limpopo | 561 | 78.61 | 13.34 | Seasonal | Savanna |
| Selati | S23.9800, E30.7166 | South Africa, Limpopo | 782 | 72.64 | 11.63 | Seasonal | Savanna |
| Rietspruit Game Reserve | S24.3978, E30.9310 | South Africa, Limpopo | 690 | 75.59 | 8.59 | Seasonal | Savanna |
| AmaKhosi Private Game Reserve | S27.6592, E31.6376 | South Africa, KwaZulu-Natal | 737 | 93.41 | 24.95 | Aseasonal | Savanna |
| Mkuze Falls Private Game Reserve | S27.5192, E31.6407 | South Africa, KwaZulu-Natal | 736 | 101.95 | 25.51 | Aseasonal | Savanna |
| uMhkuze Game Reserve | S27.6500, E32.2500 | South Africa, KwaZulu-Natal | 737 | 78.69 | 24.08 | Aseasonal | Savanna |

Table A.1 Continued

| **Study site** | **Co-ordinates** | **Country, Province** | **Average annual rainfall (mm)** | **Average rainfall October-March (mm)** | **Average rainfall April-September (mm)** | **Rainfall seasonality** | **Biome** |
| --- | --- | --- | --- | --- | --- | --- | --- |
| Hopewell Private Game Reserve | S33.6269, E26.1044 | South Africa, Eastern Cape | 565 | 39.39 | 33.91 | Aseasonal | Savanna |
| Kwandwe Private Game Reserve | S33.1494, E26.5263 | South Africa, Eastern Cape | 435 | 51.29 | 24.61 | Aseasonal | Albany Thicket |
| Phinda Private Game Reserve | S27.8230, E32.3400 | South Africa, KwaZulu-Natal | 875 | 81.54 | 28.38 | Aseasonal | Savanna |
| Thanda Private Game Reserve | S27.8854, E32.1845 | South Africa, KwaZulu-Natal | 857 | 88.07 | 28.58 | Aseasonal | Savanna |
| Manyoni Private Game Reserve | S27.8054, E32.0345 | South Africa, KwaZulu-Natal | 722 | 89.68 | 27.28 | Aseasonal | Savanna |
| Hluhluwe-iMfolozi Game Reserve | S28.1454, E32.0145 | South Africa, KwaZulu-Natal | 660 | 97.08 | 33.79 | Aseasonal | Savanna |
| Amakhala Private Game Reserve | S33.5102, E26.1544 | South Africa, Eastern Cape | 475 | 40.39 | 29.91 | Aseasonal | Albany Thicket |
| Mount Camdeboo Private Game Reserve | S32.2763, E24.8966 | South Africa, Eastern Cape | 348 | 48.49 | 19.35 | Aseasonal | Albany Thicket |
| Samara Private Game Reserve | S32.4430, E24.7633 | South Africa, Eastern Cape | 315 | 33.06 | 13.33 | Aseasonal | Albany Thicket |

Table A.1 Continued

| **Study site** | **Co-ordinates** | **Country, Province** | **Average annual rainfall (mm)** | **Average rainfall October-March (mm)** | **Average rainfall April-September (mm)** | **Rainfall seasonality** | **Biome** |
| --- | --- | --- | --- | --- | --- | --- | --- |
| Sanbona Private Game Reserve | S33.7233, E20.6152 | South Africa, Western Cape | 351 | 24.92 | 26.14 | Aseasonal | Fynbos |
| Shamwari Private Game Reserve | S33.4670, E26.0504 | South Africa, Eastern Cape | 500 | 35.40 | 24.77 | Aseasonal | Albany Thicket |
| Lalibela Private Game Reserve | S33.4705, E26.2547 | South Africa, Eastern Cape | 611 | 43.20 | 33.19 | Aseasonal | Albany Thicket |
| Mountain Zebra National Park | S32.1833, E25.4367 | South Africa, Eastern Cape | 602 | 45.53 | 12.41 | Aseasonal | Grassland |
| Addo Elephant National Park | S33.1944, E25.4636 | South Africa, Eastern Cape | 421 | 37.71 | 16.71 | Aseasonal | Albany Thicket |
| Garden Route Game Lodge | S34.2014, E21.6361 | South Africa, Western Cape | 444 | 37.33 | 39.14 | Aseasonal | Fynbos |
| Tswalu Private Game Reserve | S27.2250, E22.4111 | South Africa, Northern Cape | 331 | 37.05 | 6.17 | Seasonal | Savanna |
| Liwonde National Park | S14.8441, E35.3466 | Malawi | 953 | 138.18 | 8.25 | Seasonal | Savanna |
| Namibia farmland | S20.7297,  E17.2757 | Namibia | 385 | 54.14 | 6.00 | Seasonal | Savanna |
| Serengeti National Park | S02.3333, E34.8333 | Serengeti | 829 | 82.62 | 63.49 | Aseasonal | Savanna |
